# Extradural Hematoma Following Low-Order Domestic Blast Injury From a Pressure Cooker: A Case Report and Literature Review

**DOI:** 10.7759/cureus.103641

**Published:** 2026-02-15

**Authors:** Sri Hari Babu Sunkari, Navaneeth Pattereth, Ajay A

**Affiliations:** 1 Department of Trauma and Emergency, All India Institute of Medical Sciences, Nagpur, Nagpur, IND

**Keywords:** craniotomy, domestic blast injury, extradural hematoma, pressure cooker explosion, traumatic brain injury

## Abstract

Low-order domestic blast injuries generally result in localized soft-tissue trauma, but intracranial hemorrhage is exceptional. Pressure cooker explosions can generate high-velocity secondary projectiles capable of causing significant blunt head injury. We report a rare case of a large occipitoparietal extradural hematoma (EDH) following a domestic pressure cooker explosion. A 56-year-old woman presented with persistent headache and vomiting after being struck by the cooker lid. Her primary survey was stable with a Glasgow Coma Scale (GCS) of 14. Cranial computed tomography (CT) showed a minimally displaced left parietal fracture with a 6.5 × 2.7 cm occipitoparietal EDH, an associated midline shift of 5.8 mm, intralesional air foci, and adjacent contusions. The patient underwent emergency craniotomy with successful evacuation of the hematoma and made an uneventful recovery. This case illustrates that low-order domestic blast injuries can still produce life-threatening intracranial damage due to secondary projectiles, highlighting the critical role of early CT imaging and timely neurosurgical intervention.

## Introduction

Blast injuries vary in severity depending on the mechanism of explosion [1]. High-order explosives generate supersonic over-pressure waves capable of causing widespread internal injuries, while low-order blasts, such as domestic pressure cooker explosions, typically lack this primary blast component. Instead, injury patterns arise from thermal exposure and high-velocity secondary projectiles propelled during device failure [1].

Pressure cooker explosions primarily cause burns, soft-tissue lacerations, and maxillofacial or ocular trauma, while intracranial injuries such as extradural hematoma (EDH) are rare because the explosive mechanism is localized [1,2]. However, metallic components such as lids or valves may behave as dangerous projectiles with significant kinetic energy capable of producing skull fractures and underlying hematomas. EDH is a neurosurgical emergency usually associated with high-impact head trauma and arterial bleeding, most commonly from the middle meningeal artery [3]. Occurrence following a domestic low-order blast is exceedingly rare. Early identification and surgical evacuation are critical to preventing rapid neurological deterioration and herniation. This report describes an uncommon case of EDH following a domestic pressure cooker explosion, emphasizing the importance of structured trauma evaluation, surveillance brain imaging, and timely operative management.

## Case presentation

A 56-year-old previously healthy female patient presented to the emergency department approximately 2-3 hours after sustaining a blunt head injury due to a domestic pressure cooker explosion while cooking at home. She reported persistent headache and a single episode of vomiting. There was no loss of consciousness or seizure activity. She had no prior history of head injury, seizures, or neurological illness. There was no history of anticoagulant or antiplatelet use, alcohol consumption, or illicit drug use. She was not on any regular medications and had no known bleeding disorders or significant family medical history.

The patient was initially assessed and managed as per the Advanced Trauma Life Support (ATLS) protocol [4]. Her airway was patent and maintainable with cervical spine stabilization. She had normal respiratory effort, oxygen saturation of 98% on room air, and no external neck or chest signs of trauma. Hemodynamics were stable with pulse 88/min and blood pressure 112/70 mmHg; peripheral pulses were intact. Neurologically, her Glasgow Coma Scale (GCS) score [5] was 14 (E3V5M6), pupils were 3 mm and briskly reactive, and capillary glucose was 126 mg/dL. She was fully exposed and examined, revealing no burns or additional injuries. Chest and pelvic radiographs were normal. The Focused Assessment with Sonography in Trauma (FAST) examination was negative.

Secondary survey revealed only a 1 × 1 cm laceration over the left temporal region, which was sutured with 3-0 Ethilon (Ethicon, Inc., Somerville, NJ, US). No facial, dental, or signs of skull base injuries were present.

**Figure 1:**
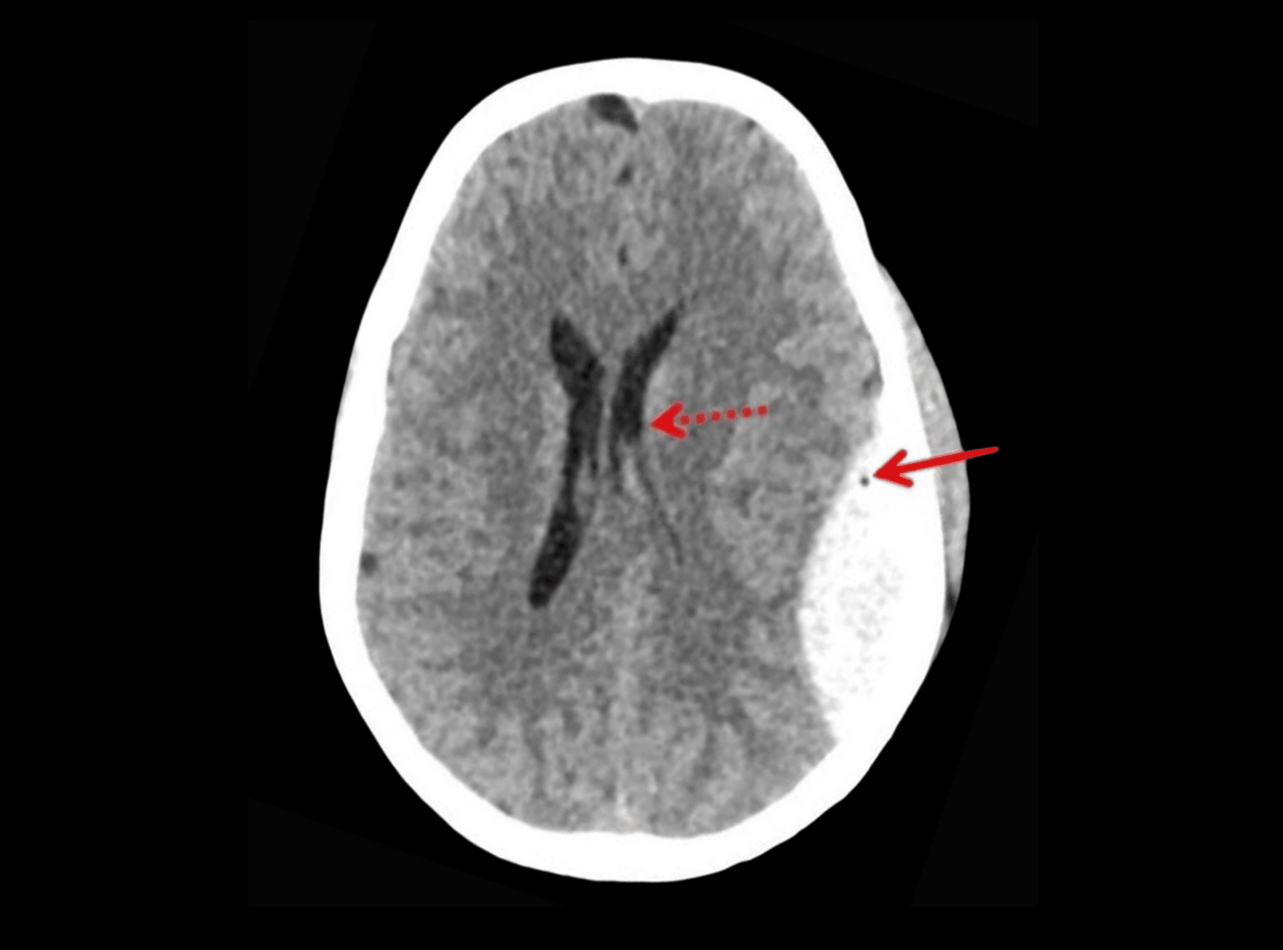
Preoperative NCCT showing left occipitoparietal EDH with air foci within (arrow) and causing compression of the ipsilateral lateral ventricle (dotted arrow). NCCT: non-contrast computed tomography; EDH: extradural hematoma

Given the mechanism-high-velocity blunt head trauma from a domestic blast-along with headache, vomiting, and GCS < 15, a non-contrast computed tomography (CT) head was performed. It showed a minimally displaced left parietal skull bone fracture extending into the temporal bone with a 6.5 × 2.7 cm occipitoparietal EDH containing air foci and a midline shift of 5.8 mm, and adjacent contusions were present, as shown in Figure 1.

**Figure 2:**
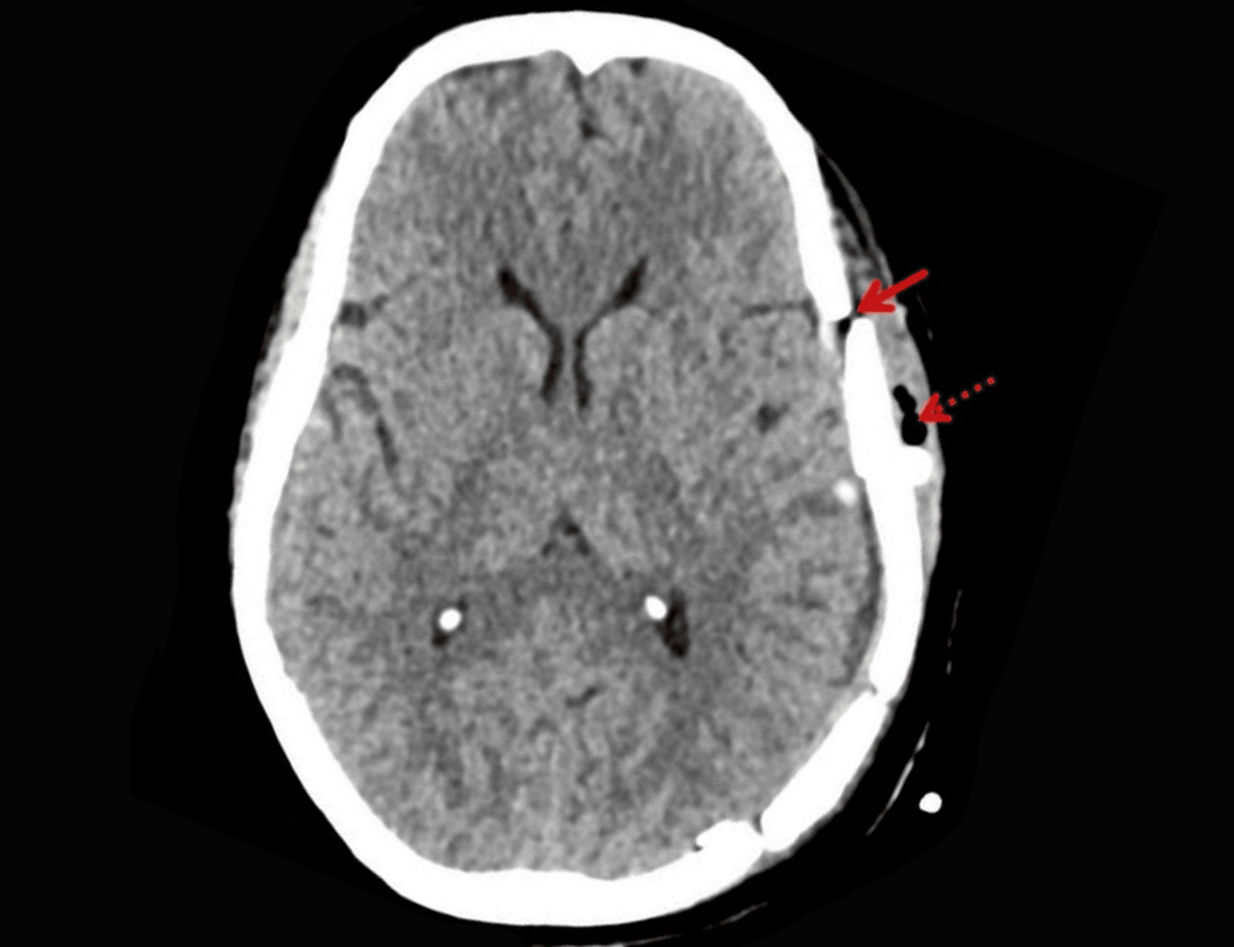
Postoperative NCCT showing complete resolution of EDH with cranioplasty defect (arrow) and subcutaneous emphysema (dotted arrow). NCCT: non-contrast computed tomography; EDH: extradural hematoma

The patient underwent emergency craniotomy and evacuation of the hematoma. Intraoperatively, extradural clots were removed, and bleeding points near the fracture line were controlled. Postoperative imaging showed a cranioplasty defect with subcutaneous emphysema and complete EDH resolution, as shown in Figure 2.

Her postoperative course was uneventful. She remained neurologically intact and was discharged on postoperative day five. At follow-up, she remained asymptomatic with no focal neurological deficits.

## Discussion

Blast injuries are categorized based on detonation velocity into high-order and low-order explosions. Low-order explosives, such as gas- or gunpowder-based devices, produce a subsonic pressure wave and primarily cause injury via secondary (shrapnel), tertiary (impact), and quaternary (burns) mechanisms. While they lack the supersonic pressure wave characteristic of high-order explosives, this case demonstrates that low-order domestic blasts can still cause skull fractures and intracranial hemorrhage [1].

Traumatic brain injury (TBI) from domestic accidents is increasingly being recognized in the literature. Domestic environments, once thought to be low-risk settings, are emerging as sources of potentially serious injuries, including intracranial hemorrhage. In one large retrospective series, domestic injuries accounted for nearly 25% of EDH cases requiring surgical evacuation [2]. This case adds to a small but growing body of evidence showing that household appliances, including pressure cookers, can result in significant neurotrauma.

Blast-related EDH in a domestic setting is extremely rare. Previous reports include injuries caused by cooker regulator valves [3], whistle-related penetrating ocular trauma [6], burns from lid blowouts [7], burns and mandible fractures due to pressure cooker explosions [8], and contusions after being hit by the pressure regulator of a pressure cooker [9]. A case series from Sri Lanka described seven cases of facial burns due to pressure cooker use, further supporting the risk of domestic cooking appliances [10].

In another rare instance, some reported a case of TBI in a child following a pressure cooker explosion, emphasizing that children and adults alike are at risk [11]. Similarly, isolated reports have described penetrating ocular and craniofacial trauma from pressure cookers, indicating a range of possible injury patterns [12,13]. Table 1 shows a comprehensive overview of previously reported head or intracranial injuries associated with pressure cooker explosions.

**Table 1:** Previously reported head or intracranial injuries associated with pressure cooker explosions.

Authors (year)	Number of cases/age	Characteristics of head or intracranial injury	Management	Outcome
Gupta et al. (2014) [3]	1 case/adult	Penetrating transorbital craniocerebral injury caused by a pressure cooker component	Surgical exploration and removal of foreign body	Favorable neurological recovery
Das et al. (2021) [9]	2 cases/adults	Rare head injuries including intracranial involvement following pressure cooker explosions	Surgical and conservative management based on injury severity	Both patients recovered
Calderon-Miranda et al. (2016) [11]	1 case/child	Traumatic brain injury following pressure cooker explosion	Conservative management with close neurological monitoring	Complete recovery
Esposito et al. (2018) [14]	1 case/adult	Polytrauma including head injury secondary to unintentional pressure cooker explosion	Multidisciplinary trauma management including surgical intervention	Survived with good functional outcome
Kumar et al. (2018) [15]	1 case/adult	Blunt head injury with skull fracture due to pressure cooker blast	Surgical management	Neurological improvement
Present case (2024)	1 case/56-year-old female	Minimally displaced parietotemporal skull fracture with large occipitoparietal extradural hematoma and associated midline shift	Emergency craniotomy and hematoma evacuation	Uneventful recovery; discharged neurologically intact

Management of epidural hematoma depends on hematoma size, neurological status, and radiological findings. Small, asymptomatic hematomas without significant mass effect may be managed conservatively with close neurological and radiological monitoring. However, surgical evacuation is recommended in patients with neurological deterioration, hematoma thickness greater than 15 mm, midline shift exceeding 5 mm, or a GCS score less than 9. In the present case, the presence of a significant midline shift with clinical symptoms warranted urgent surgical intervention. Recent studies have demonstrated favorable outcomes with timely operative management when indicated [16].

This case underscores the importance of mechanism-based assessment in emergency medicine. Domestic pressure cooker explosions represent low-order blast injuries that may cause significant intracranial pathology despite minimal external signs. Awareness of such mechanisms can prompt early imaging and timely intervention, thereby preventing secondary brain injury and improving outcomes.

## Conclusions

Domestic pressure cooker explosions, though classified as low-order blasts, can result in serious intracranial injuries such as skull fractures and intracranial hematomas. Timely imaging and surgical management are essential for optimal recovery. This case also highlights the need for improved appliance safety standards and greater public awareness to help prevent such potentially life-threatening injuries in home settings.

## References

[1] Gordon W, Kuhn K, Staeheli G, Dromsky D (2015). Challenges in definitive fracture management of blast injuries. Curr Rev Musculoskelet Med.

[2] Jamieson KG, Yelland JD (1968). Extradural hematoma. Report of 167 cases. J Neurosurg.

[3] Gupta OP, Roy K, Ghosh S, Tripathy P (2014). An unusual penetrating transorbital craniocerebral injury. Indian J Neurotrauma.

[4] American College of Surgeons (2025). American College of Surgeons. Advanced trauma life support (ATLS) student course manualChicago (IL). Advanced Trauma Life Support (ATLS) Student Course Manual.

[5] Jain S, Margetis K, Iverson LM (2025). Glasgow Coma Scale. StatPearls [Internet].

[6] Thapa DK, Vyas S, Saiju R, Rajbhandari P, Pant B (2019). An unusual case of penetrating ocular trauma with a pressure cooker whistle. Eastern Green Neurosurgery.

[7] Sandhir RK, Sandhir M (1992). Accidental pressure cooker lid blow-out. Burns.

[8] Gundeslioglu AO, Yenidunya MO (2010). Burn and mandible fracture due to pressure cooker explosion. J Craniofac Surg.

[9] Das JM, Pokharel A, Sapkota R, Mishra M, Babu Aryal A (2018). Case report: two cases of rare head injuries from Nepal. F1000Res.

[10] Perera VA, Karunadasa K, Perera C (2012). A case series of domestic pressure cooker burns. Ceylon Med J.

[11] Calderon-Miranda WG, Escobar-Hernandez N, Moscote-Salazar LR (2016). Traumatic brain injury due to pressure cooker explosion in a child: case report. Romanian Neurosurgery.

[12] Sarkar S, Modi S, Seth AK, Panja S (2015). An unusual transorbital penetrating injury by house-key (lock): a case report with a small review of literature. J Clin Diagn Res.

[13] Bullock MR, Chesnut R, Ghajar J (2006). Surgical management of acute epidural hematomas. Neurosurgery.

[14] Esposito M, Meyer M, Strote J (2018). Polytrauma from unintentional pressure cooker explosion: a case report. J Emerg Med.

[15] Kumar M, Harsh V, Jha S, Prakash A, Kumar A, Sahay CB (2018). An unusual case of head injury by pressure cooker explosion. International Journal of Medical Science and Innovative Research (IJMSIR).

[16] Gok H, Celik SE, Yangi K, Yavuz AY, Percinoglu G, Unlu NU, Goksu K (2023). Management of epidural hematomas in pediatric and adult population: a hospital-based retrospective study. World Neurosurg.

